# Malproduction of endogenous hydrogen gas in COVID-19

**DOI:** 10.3389/fcimb.2022.924832

**Published:** 2022-07-28

**Authors:** Sergej M. Ostojic

**Affiliations:** ^1^ Department of Nutrition and Public Health, University of Agder, Kristiansand, Norway; ^2^ Applied Bioenergetics Lab, Faculty of Sport and PE, University of Novi Sad, Novi Sad, Serbia

**Keywords:** oxidative stress, gasotransmitters, molecular hydrogen, COVID-19, gut microbiota

## Abstract

The molecular footprints of COVID-19 occur everywhere, even reaching the family of biologically active gases and gasotransmitters. Besides nitric oxide and hydrogen sulfide, COVID-19 might also alter the homeostasis of dihydrogen (H_2_), another gaseous bioactive molecule produced endogenously by the human gut bacteria. Many studies have shown various alterations of the gut microbiota in patients with coronavirus disease 2019, including the lower abundance of hydrogen-producing bacteria that could instigate the shortage of hydrogen output. Since dihydrogen has many important bioactivities, including cytoprotective, antioxidant, anti-inflammatory, and antiapoptotic, its malproduction in COVID-19 might contribute to the disease progression and severity. On the other hand, replenishing dihydrogen by exogenous administration could be beneficial in COVID-19 for both patient- and clinical-reported outcomes. Assessing low dihydrogen along with H_2_ supplementation to restore normal levels could be thus combined *via* theranostic approaches to aid COVID-19 diagnosis and treatment.

The coronavirus disease 2019 (COVID-19) has thus far attracted the unprecedented attention of the biomedical research community that has been trying to tackle its transmission, pathogenesis, diagnosis, and treatment. From the benchside standpoint, the molecular signatures of COVID-19 occur everywhere (Li et al., 2021), with new data putting forward not only potential therapeutic targets but also biomarkers for understanding the pathogenesis of the disease. A recent study demonstrated low availability of key endogenous gasotransmitters, nitric oxide (NO), and hydrogen sulfate (H_2_S), in a cohort of COVID-19 patients (Dominic et al., 2021). The authors found significantly reduced NO and H_2_S concentrations in blood samples of COVID-19 patients compared with healthy controls, and free sulfide levels were highly predictive of COVID-19 infection based on reduced turnover. This strongly suggests a dysregulation of normal gasotransmitter homeostasis during the disease, either due to impaired production and/or enhanced usage of critical bioactive endogenous gaseous signaling molecules. The biodynamics of another internally produced gas, molecular hydrogen (H_2_, dihydrogen), might also be compromised during the disease, either due to the reduced synthesis secondary to gut dysbiosis, or increased consumption from scavenging of reactive oxygen species (ROS).

Several recent studies have demonstrated the reduction in microbial diversity after the disease, with COVID-19 gut microbiota composition reflecting disease severity and dysfunctional immune responses (Gu et al., 2020; Zuo et al., 2020). Interestingly, a COVID-19-driven dysbiosis appears to be characterized by the low abundance of hydrogen-producing bacteria, including *Faecalibacterium prausnitzii*, *Eubacterium rectale*, and bifidobacteria (Yeoh et al., 2021). Hydrogen-producing bacteria use two enzyme systems to generate H2 during fermentation, including hydrogenases and nitrogenase, with total dihydrogen excretion rates of up to 200 ml per 24 h expired in the breath of healthy subjects (Christl et al., 1992), and this might be compromised during COVID-19. Although the production of H_2_ has not been evaluated in the above studies, hydrogenotrophic strains were under-represented in COVID-19 patients and remained low in samples collected up to 30 days after disease resolution. In line with this, our group found lower levels of H_2_ in breath samples of COVID-19 patients naïve to antibiotic therapy as compared to healthy subjects (Ostojic et al. unpublished data), suggesting inadequate production of gut microbiota-derived H_2_. Dihydrogen levels were measured with a gas-specific electrochemical sensor, yet these preliminary findings remain highly speculative due to the small number of subjects, and should be corroborated in a well-sampled clinical trial. A possible COVID-19-driven hydrogen shortfall may perhaps happen due to SARS-CoV-2 infection-driven aggressive inflammatory responses in enterocytes *via* highly expressed angiotensin-converting enzyme 2 (ACE2) and transmembrane protease serine 2 (TMPRSS2) receptors (Zhang et al., 2021), that eventually affect microbial ecology and dihydrogen production. Alternatively, the virus could directly attack the host microbiota and alter normal production of gut bacteria-derived metabolites or components, including short-chain amino acids and/or hydrogen (Wang et al., 2022). Dihydrogen acts as a versatile and selective antioxidant (Ohsawa et al., 2007). A possible consequence of COVID-19-induced H_2_ shortage could thus lead to deprived antioxidant defense and ROS-originated damage, which have been already recognized as key players in the disease pathogenesis and progression (Delgado-Roche and Mesta, 2020). Interestingly, hydrogen deficiency states have been suggested in various conditions, including infectious diseases, nutritional deficiencies, and metabolic conditions [for a review, see Ref (Williams and Ramsden, 2007).

Unsurprisingly, the provision of exogenous dihydrogen in COVID-19 has been proven beneficial in several preliminary trials, with enteral or inhalational H_2_ ameliorating clinical features of the disease and biomarkers of inflammation and oxidative stress (for a detailed review, see Ref (Alwazeer et al., 2021).). A seminal early report by China’s National Health Commission and the Chinese Center for Disease Control and Prevention disclosed a rather exotic ratio of dihydrogen and oxygen delivered in a 2:1 ratio (66% and 33%, respectively) as the composition of the gas inhalation mixture for COVID-19 treatment (National Health Commission of the People’s Republic of China). A multicenter randomized clinical trial verified the efficacy and safety of dihydrogen inhalation in patients with COVID-19 (Guan et al., 2020), demonstrating that the novel treatment reduces dyspnea, chest pain, and cough, improves resting oxygen saturation, and promotes disease recovery. Another trial demonstrated that the concentration of protective proteins (including dermcidin, an antibiotic and proteolytic protein) in exhaled breath condensate increased significantly after hydrogen supplementation in volunteers who had recovered from COVID-19 (Ryabokon et al., 2021). Finally, hydrogen-water alleviates disease-related fatigue and inflammation biomarkers in middle-aged COVID-19 patients with mild to moderate disease severity (Milovancev et al., 2022). Although no mechanistic studies have been conducted so far, exogenous gas could re-establish dihydrogen homeostasis jeopardized by SARS-CoV-2 infection and reduce lung damage by acting as a cytoprotective biomolecule. Besides neutralizing toxic ROS, potential cellular and molecular targets of dihydrogen in COVID-19 include neutrophils, macrophages, and cytokines (Li et al., 2021). In addition, other abilities of dihydrogen in the context of inflammation/infection management should be considered, including its role as an anti-apoptotic bioactive and signaling molecule that can upregulate critical pathways in immune cells (Itoh et al., 2009).

While the low availability of gut bacteria-generated dihydrogen in COVID-19 might be an important advancement, understanding its contribution to the disease progression and clinical treatment remains complicated. The disease-driven shortage of hydrogen has to be addressed in terms of COVID-19 severity and duration and across various demographic and ethnic populations, while controlling for environmental and genetic factors that could interfere with hydrogenotrophic bacteria diversity. Measuring dihydrogen levels in breath is not a routine practice in COVID-19 monitoring at this moment, although the method is non-invasive and relatively straightforward, preferably followed by blood dihydrogen assaying. Early trials found that dihydrogen is safe and effective in COVID-19, yet this innovative therapy must move to advanced clinical trials, while adhering to strict regulatory requirements, and possibly earn FDA authorization. Dihydrogen theranostics in COVID-19 remain to be further studied.

## Data Availability Statement

The original contributions presented in the study are included in the article/supplementary material. Further inquiries can be directed to the corresponding author.

## Author Contributions

SO discussed this issue and wrote the paper; and had primary responsibility for final content. The author read and approved the final manuscript.

## Conflict of Interest

The author declares that the research was conducted in the absence of any commercial or financial relationships that could be construed as a potential conflict of interest.

## Publisher’s Note

All claims expressed in this article are solely those of the authors and do not necessarily represent those of their affiliated organizations, or those of the publisher, the editors and the reviewers. Any product that may be evaluated in this article, or claim that may be made by its manufacturer, is not guaranteed or endorsed by the publisher.

## Abbreviations

ACE2, angiotensin-converting enzyme 2; COVID-19, Coronavirus disease 2019; H_2_, molecular hydrogen; H_2_S, hydrogen sulfate; NO, nitric oxide; ROS, reactive oxygen species; TMPRSS2, transmembrane protease serine 2.
